# Research progress on bleeding risk assessment models in anticoagulant therapy

**DOI:** 10.3389/fcvm.2025.1645823

**Published:** 2025-11-11

**Authors:** Li Sen, Xiong Kangpin, Liu Yihui

**Affiliations:** 1 Department of Pharmacy, Union Hospital, Tongji Medical College, Huazhong University of Science and Technology, Wuhan, Hubei, China; 2 Department of Pharmacy, Wuhan Asia Heart Hospital, Wuhan University of Science and Technology, Wuhan, Hubei, China

**Keywords:** anticoagulant therapy, bleeding risk assessment models, atrial fibrillation, venous thromboembolism, non-vitamin K antagonist oral anticoagulants

## Abstract

Balancing thromboembolic prevention against bleeding complications remains a critical challenge in anticoagulant therapy. While established bleeding risk assessment models (RAMs) such as HAS-BLED and HEMORR_2_HAGES were initially developed for warfarin-treated patients, their applicability to non-vitamin K antagonist oral anticoagulant (NOAC) users and venous thromboembolism (VTE) populations remained uncertain. This review synthesized recent advancements in bleeding risk stratification for atrial fibrillation (AF) and VTE patients, focusing on model performance, drug-specific adaptations, and emerging biomarker-driven approaches. For AF patients, traditional RAMs (HAS-BLED, HEMORR_2_HAGES, ATRIA) demonstrated moderate predictive accuracy (AUC: 0.55–0.74) in NOAC cohorts, with HEMORR_2_HAGES showing superior discrimination in certain studies. The biomarker-integrated ABC (incorporating GDF-15, troponin, hemoglobin) and the NOAC-specific DOAC score, have shown improved risk stratification, with the latter demonstrating a higher C-statistic than HAS-BLED. In VTE populations, the IMPROVE (AUC: 0.62–0.73) effectively identified high-risk medical inpatients, while the RIETE (major bleeding incidence: 0.1%–6.2%) and EINSTEIN (C-statistic: 0.68–0.74) addressed dynamic risks during anticoagulation. However, heterogeneity in validation cohorts, endpoint definitions (e.g., ISTH vs. TIMI criteria), and static risk factor selections limited cross-model generalizability. Current RAMs exhibited variable performance across anticoagulant regimens and clinical contexts highlighting the need for next-generation models that integrate dynamic risk modifiers (e.g., transient anemia, antiplatelet use) and biomarker-based approaches. While NOAC-specific tools such as the DOAC may be more appropriate for AF patients, context-adapted models like IMPROVE and RIETE are better suited for VTE populations. Future research should prioritize real-world validation, machine learning integration, and the standardization of bleeding definitions to advance precision anticoagulation strategies.

## 1 Introduction

Anticoagulant therapy remains the cornerstone for preventing thromboembolic events in patients with atrial fibrillation (AF) and venous thromboembolism (VTE). Globally, AF affects over 37.6 million individuals, while the annual incidence of VTE exceeds 10 million cases, both conditions carrying substantial morbidity and mortality burdens (1, 2). Despite their proven efficacy, anticoagulants inevitably increased the risk of bleeding, with major events such as intracranial hemorrhage (ICH) and gastrointestinal (GI) bleeding leading to a 30-day mortality rate of 11.3% and long-term disability among survivors (3). Notably, the highest incidence of major bleeding (2.0%–3.5% annually) occurred during the initial 3-month “vulnerable phase” of therapy, a critical period when bleeding-related anticoagulation discontinuation may paradoxically increase thromboembolic recurrence by 4.6-fold (4, 5).

The clinical imperative to balance thromboprophylaxis against hemorrhagic complications necessitates precise risk stratification. While individual risk factors, such as advanced age, renal impairment, or prior bleeding, have limited predictive value, integrated risk assessment models (RAMs) have emerged as essential tools for personalized decision-making (6). Historically, models like HAS-BLED and HEMORR2HAGES were developed and validated in warfarin-treated cohorts, reflecting the therapeutic landscape before the advent of non-vitamin K antagonist oral anticoagulants (NOACs) (7, 8). However, with NOACs now being prescribed to more than 70% of AF patients in high-income countries, their widespread adoption has introduced novel pharmacokinetic profiles and drug-drug interactions, posing challenges to the generalizability of existing models (9, 10). Recent meta-analyses suggested discordant performance of traditional RAMs in NOAC users, with area-under-curve (AUC) values ranging from 0.55 to 0.73, underscoring the need for drug-specific validation and recalibration (11, 12).

Furthermore, evolving insights into dynamic risk modifiers (e.g., transient anemia, fluctuating blood pressure, or concomitant antiplatelet use) and biomarker-driven approaches [e.g., growth differentiation factor-15 (GDF-15) or high-sensitivity troponin] have catalyzed the development of next-generation RAMs (13, 14). These models aimed to address the limitations of static clinical scores by incorporating real-time physiological data and machine learning algorithms. Nevertheless, heterogeneity in study designs, endpoint definitions (e.g., ISTH vs. TIMI bleeding criteria), and population characteristics complicated cross-model comparisons and hindered clinical implementation (15).

Given the growing importance of precision medicine in anticoagulation management, this review aims to summarize the latest advancements in bleeding risk assessment models for AF and VTE patients undergoing anticoagulation therapy. Evidence was synthesized from a non-systematic literature search conducted in PubMed and Web of Science up to 2024. Search terms included combinations of “anticoagulant”, “bleeding risk”, “risk assessment model”, “atrial fibrillation”, “venous thromboembolism”, and specific model names (Table 1). Studies were selected based on clinical relevance and methodological rigor, with priority given to those that described widely implemented clinical scores, were derived from large cohort studies or randomized controlled trials, and included data from external validation.

**Table 1:** Evidence grade of bleeding risk assessment models included in this review.

Model	Study type	Evidence grade
HAS-BLED	Cohort study	Moderate
ORBIT	Cohort study	Moderate
ATRIA	Cohort study	Moderate
HEMORR_2_HAGES	Retrospective analysis	Low
OBRI	Cohort study	Low
Shireman	Cohort study	Low
ABC	Research on predictive models	High
DOAC	Randomized controlled trial	High
Kuijer	Cohort study	Low
RIETE	Cohort study	Moderate
EINSTEIN	Randomized controlled trial	High
Hokusai	Cohort study	Moderate
Seiler	Cohort study	Moderate
VTE-BLEED	Research on predictive models	Moderate

By critically evaluating the strengths and limitations of established and emerging models, this review aims to provide an updated synthesis of bleeding risk stratification strategies, highlight gaps in model applicability across therapeutic contexts, and discuss future directions for developing more dynamic, biomarker-integrated, and individualized prediction tools in anticoagulant management.

## 2 Bleeding risk assessment in AF patients

### 2.1 The bleeding risk assessment models for AF patients

#### 2.1.1 HAS-BLED

This model includes risk factors such as hypertension (1 point), abnormal liver or renal function (1 point each), stroke (1 point), history of bleeding or predisposition (1 point), unstable INR (1 point), elderly age (1 point), and use of medications or alcohol abuse (1 point each). A total score of ≥3 indicates a high bleeding risk.

#### 2.1.2 ORBIT

This model assessed anemia (1 point), prior bleeding history (2 points), renal impairment (2 points), age >75 years (1 point), and used of antiplatelet agents (1 point). A score of 0–2 is considered low risk, 3 is intermediate risk, and ≥4 is high risk.

#### 2.1.3 ATRIA

The model assigned points for anemia (3 points), severe renal disease (glomerular filtration rate <30 ml/min or dialysis-dependent, 3 points), age ≥75 years (2 points), previous bleeding (1 point), and hypertension (1 point). Scores of 0–3 indicate low risk, 4 is intermediate risk, and 5–10 is high risk.

#### 2.1.4 HEMORR_2_HAGES

This model considered liver or kidney disease, alcohol abuse, malignancy history, age >75 years, thrombocytopenia or platelet dysfunction, rebleeding risk, hypertension, anemia, genetic factors (CYP2C9), falls, and stroke. A score of 0–1 is low risk, 2–3 is intermediate risk, and ≥4 is high risk.

#### 2.1.5 OBRI

This model evaluated age ≥65 years, history of stroke, history of gastrointestinal ulcer bleeding, and any of the following comorbidities: recent myocardial infarction, hematocrit <30%, creatinine >1.5 mg/L, or diabetes history. Each factor is assigned 1 point. A total score of 0 is low risk, 1–2 is intermediate risk, and ≥3 is high risk.

#### 2.1.6 Shireman

This model included age ≥70 years, female sex, previous bleeding, recent bleeding, alcohol or drug abuse, diabetes, anemia, and use of antiplatelet drugs. Unlike integer-based scores, the Shireman score produces a continuous risk value based on regression coefficients. In validation studies, scores <1.07 indicated low risk (corresponding to 0.9% annual bleeding risk), 1.07–2.19 indicated intermediate risk (2.0% annual risk), and >2.19 indicated high risk (5.4% annual risk).

#### 2.1.7 ABC

Unlike traditional models based on clinical risk factors, the ABC incorporated biomarkers (growth differentiation factor-15, high-sensitivity troponin T, hemoglobin) alongside age and bleeding history. The model generates a continuous risk prediction rather than categorical risk groups. The biomarker levels are weighted and combined with clinical factors to estimate the absolute probability of major bleeding at 1 and 3 years, allowing for more precise risk stratification beyond the traditional low, medium, high classification.

#### 2.1.8 DOAC

Developed specifically for NOAC users, this model was derived from a secondary analysis of the RE-LY trial. It evaluated age, creatinine clearance/glomerular filtration rate, low body weight, history of stroke/transient ischemic attack/embolism, diabetes, hypertension, antiplatelet use, NSAID use, liver disease, and previous bleeding. Each additional point increased major bleeding risk by 48.7% (95% CI, 38.9%-59.3%; *P* < 0.001). The model has demonstrated superior predictive performance compared to the HAS-BLED (16).

To provide a clear overview and facilitate comparison, the key characteristics of these AF-specific bleeding risk assessment models were summarized in Table 2.

**Table 2:** Summary of major bleeding risk assessment models in atrial fibrillation (AF) patients.

Model	Original study design/validation cohort	Sample size (*n*)	Main predictive factors	Risk stratification	Major bleeding incidence (%)	C-statistic (AUC)	Key features
HAS-BLED	Derived from the Euro Heart Survey; external validation in multiple AF cohorts	OAC: 2,115 Aspirin/Clopidogrel: 828 Neither: 352	Hypertension, Abnormal renal/liver function, Stroke, Bleeding history, Labile INR, Elderly (≥65), Drugs/alcohol use	Low risk: 0–2 points High risk: ≥3 points	OAC: 1.75% Aspirin/Clopidogrel:0.97% Neither: 1.42%	OAC: 0.72 Aspirin/Clopidogrel: 0.91 Neither: 0.85	Simple and widely recommended in guidelines; moderate performance in NOAC users
ORBIT	Derived and validated from the ORBIT-AF registry	Warfarin: 6,929 Dabigatran: 482	Age ≥75, Reduced Hemoglobin/hematocrit, Bleeding history, Insufficient kidney function, Treatment with antiplatelets	Low risk: 0–2 points Intermediate: 3 points High risk: ≥4 points	4%	0.67 (0.64–0.69)	Better short-term prediction; may be preferable for NOAC-treated patients
ATRIA	Kaiser Permanente cohort; prospective validation	Warfarin: 9,186	Anemia, Severe renal disease, Age ≥75, Prior bleeding, Hypertension	Low risk: 0–3 points Intermediate: 4 points High risk: 5–10 points	Low risk: 0.8% Intermediate: 2.6% High risk: 5.8%	Warfarin: 0.74 NOAC: 0.58–0.61	Good in warfarin cohorts; limited performance in NOAC users
HEMORR_2_HAGES	Retrospective cohort from US Veterans database; external validation in AF cohorts	Warfarin: 1,604 Aspirin: 660 Neither: 1,527	Hepatic/renal disease, Ethanol abuse, Malignancy, Older age, Reduced platelet/function, Rebleeding, Hypertension, Anemia, Genetic factors, Excessive fall risk, Stroke	Low risk: 0–1 point Intermediate: 2–3 points High risk: ≥4 points	Warfarin: 4.9% Aspirin: 5.9% Neither: 4.1%	Warfarin: 0.67 Aspirin: 0.72 Neither: 0.66	Strong predictor but complex; best performer in some NOAC studies
OBRI	Derived from retrospective warfarin-treated outpatient cohort (U.S. Veterans Affairs); later prospectively validated in VTE and AF populations	Warfarin: 820	Age ≥65 years, History of stroke, History of gastrointestinal bleeding or peptic ulcer, and ≥1 of the following: recent myocardial infarction, hematocrit <30%, serum creatinine >1.5 mg/dl, or diabetes	Low risk: 0 points Intermediate: 1–2 points High risk: ≥3 points	6.5%	0.72	Simple but limited predictive ability; mainly for outpatient assessment
Shireman	Retrospective warfarin-treated cohort (Medicare database)	Warfarin: 26,345	Age ≥75, History of GI bleeding, Anemia, Recent hospitalization, Prior stroke	Low risk: <1.07 points Intermediate: 1.07–2.19 points High risk: >2.19 points	Low risk: 0.9% Intermediate: 2.0% High risk: 5.4%	N/A	Provides continuous risk estimate rather than categorical groups
ABC	ARISTOTLE & RE-LY biomarker substudies	ARISTOTLE, apixaban vs. warfarin: 14,537; RELY, dabigatran vs. warfarin: 8,468	Age, Biomarkers (GDF-15, troponin, hemoglobin), Clinical bleeding history	Low risk: ≤3 points Intermediate: 4–7 points High risk: ≥8–9 points	N/A	0.71 (0.68–0.73)	Incorporates biomarkers for dynamic and individualized risk prediction
DOAC	Derived from RE-LT trail, GARFIELD-AF registry; external validation in 12,296 NOAC-treated patients	RELY: 5,684, GARFIELD-AF: 12,296	Age, Renal function, Liver disease, Antiplatelet/NSAID use, Anemia, Cancer, Polypharmacy	Very low risk: 0–3 points Low risk 4–5 points Intermediate: 6–7 points High risk: 8–9 points Very high risk: ≥10 points	RE-LY: 6.8% GARFIELD-AF: 1.1%	RE-LY: DOAC 0.72 (0.71–0.74) HAS-BLED 0.6 (0.58–0.62); GARFIELD-AF: DOAC 0.71 (0.67–0.75) HAS-BLED 0.66 (0.62–0.71)	NOAC-specific; accounts for drug interactions and renal function; superior to HAS-BLED

N/A, indicates that specific values were not provided in the referenced studies.

### 2.2 Comparative analysis of bleeding risk models for non-valvular atrial fibrillation (NVAF) patients

The effectiveness of bleeding risk scores is often evaluated using the area under the receiver operating characteristic curve (AUC or C-statistic). The AUC curve serves as the primary and objective metric to determine the discriminative performance of each model. Typically, the AUC ranges from 0.5 to 1.0, with values closer to 1 indicating better discrimination. For binary outcomes such as bleeding vs. non-bleeding, the C-statistic is numerically equivalent to the AUC. From a practical perspective, these discrimination metrics help clinicians gauge how well a model distinguishes between patients who will and will not experience bleeding events. A C-statistic of 0.5 indicates no discriminative power (equivalent to random chance), 0.7–0.8 reflects acceptable predictive ability, and >0.8 represents excellent performance. Thus, while high AUC values support the model's reliability for risk identification, clinical decision-making should integrate these quantitative results with individualized patient assessment and clinical context.

In warfarin users, the AUC values for HAS-BLED, HEMORR_2_HAGES, and ATRIA were 0.72, 0.69, and 0.74, respectively. The HAS-BLED has shown AUC values as high as 0.91 in certain populations, indicating superior discrimination, and it remains widely recommended in clinical guidelines (17–19). However, most models were developed and validated in patients receiving warfarin and heparin, making their applicability to NOAC users uncertain.

Recent studies have explored optimal bleeding risk models for NOAC-treated NVAF patients. Tchen et al. (17) retrospectively analyzed 126 patients and found that the AUC values for HAS-BLED, HEMORR_2_HAGES, and ATRIA in predicting major bleeding among NOAC users were 0.645, 0.675, and 0.580, respectively, with HEMORR_2_HAGES performing best. However, the study was limited by its small sample size. Campos-Staffico et al. (20) analyzed 2,364 patients receiving rivaroxaban or apixaban and found that at 100 days, ORBIT and ATRIA had the highest C-statistics (0.62 and 0.61, respectively), while all scores had C-statistics <0.60 at 2,100 days, suggesting that ORBIT may be preferable for NOAC users. A large multicenter prospective cohort study (CACAO) followed 3,082 patients for one year and found that among six bleeding risk models, only HEMORR_2_HAGES showed moderate predictive ability for major bleeding (C-statistics 0.66 and 0.57) (21).

Wang et al. (22) valuated the predictive ability of the ABC for major bleeding risk in Chinese AF patients receiving oral anticoagulation (OAC) therapy in a real-world setting. The study included 2,892 Chinese AF patients on OAC therapy, with a median treatment duration of 265 days for those on NOACs. Over a 3-year follow-up period, the incidence of bleeding events was 0.51 per 100 patient-years. The ABC showed a higher hazard ratio for identifying high-risk patients compared to the HAS-BLED (HR: 4.92 vs. 3.70), and it was effective in distinguishing between moderate and high-risk patients. However, it may overestimate the actual major bleeding risk. Therefore, the researchers suggested that both scores were used together in clinical practice.

To clarify the underlying structure and rationale of the models, the Shireman was originally designed to assess bleeding risk in warfarin-treated patients using a simple point-based framework. Its main parameters include age ≥75 years, prior gastrointestinal bleeding, anemia, and recent hospitalization, each assigned a weighted score that classifies patients into low, moderate, and high risk groups. This model primarily emphasizes demographic and comorbidity-related predictors. By contrast, the ABC integrates Age, Biomarkers [growth differentiation factor-15 (GDF-15), high-sensitivity troponin, and hemoglobin], and Clinical history (previous bleeding). The inclusion of biomarkers allows this model to capture subclinical physiological alterations, improving individualized and dynamic bleeding risk prediction during anticoagulation therapy.

The DOAC score, compared to previous scores, distinguishes bleeding risk differences across more age groups, thereby improving personalized risk estimation. Furthermore, this scoring system takes into account the bleeding risk variations associated with different levels of renal function, as renal impairment is a strong risk factor for bleeding. Additionally, many AF patients were concurrently taking multiple medications associated with high bleeding risk, and the DOAC score highlighted the cumulative risk for patients on multiple drugs, as well as the risk differences associated with various treatment combinations. In an external validation study involving 12,296 patients from the GARFIELD-AF registry, the DOAC score demonstrated superior performance compared to the HAS-BLED score (C-statistic: 0.71 vs. 0.66; *P* = 0.025) (16).

Overall, while the HEMORR_2_HAGES exhibited strong predictive capability, its complexity limits routine clinical use. HAS-BLED remains the most widely applied model because of its balance between simplicity and predictive power. The ABC, particularly when combined with HAS-BLED, offers improved sensitivity for identifying high-risk individuals, while the DOAC provides a more tailored and pharmacologically relevant approach for NOAC-treated patients.

## 3 Bleeding risk assessment in venous thromboembolism (VTE) patients

### 3.1 Bleeding risk assessment during preventive anticoagulation for VTE

The IMPROVE is the first bleeding risk assessment model designed specifically for VTE prevention, derived from an international observational study (23). This study included 10,866 medical patients, using Kaplan–Meier analysis to assess in-hospital bleeding incidence and multivariate regression to identify bleeding-related risk factors. Results showed a cumulative incidence of major and non-major bleeding of 3.2% within 14 days of hospitalization. The strongest independent predictors of bleeding in hospitalized patients were active gastroduodenal ulcer, prior bleeding history, and low platelet count. Other risk factors included older age, liver or renal failure, ICU admission, central venous catheterization, rheumatic diseases, malignancy, and male sex. An IMPROVE score ≥7 indicates a high risk of bleeding, suggesting that mechanical prophylaxis rather than anticoagulants should be considered.

A retrospective study validated the IMPROVE externally in 12,082 hospitalized medical patients, classifying them into low-risk (<7 points) and high-risk (≥7 points) groups (24). The overall bleeding rate within 14 days of admission was 2.6%, with major bleeding rates of 1.5% in the low-risk group vs. 3.2% in the high-risk group (OR 2.2, 95% CI: 1.6–2.9, *p* < 0.0001). The sensitivity and specificity of the IMPROVE for predicting bleeding were 34.0% and 81.5%, respectively. This study represented the largest external validation of the IMPROVE to date, confirming that a threshold of 7 points effectively identified high-risk patients for major and overall bleeding events. Another retrospective study by Wang et al. (25) assessed bleeding risk in 138 hospitalized COVID-19 patients using the IMPROVE. Nine patients (6.5%) were classified as high risk, all of whom were critically ill. Among them, six (4.3%) experienced bleeding complications after anticoagulation therapy, including mild hematuria or microscopic hematuria (32.2%), mild gastrointestinal bleeding (10.7%), moderate epistaxis (10.7%), and severe hemothorax. This study demonstrated that the IMPROVE has good predictive performance for bleeding risk in critically ill COVID-19 patients.

The IMPROVE has been externally validated in both European and Chinese populations, showing moderate to good discriminatory ability for predicting bleeding in hospitalized medical patients (26, 27). However, its utility in surgical patients remains to be further evaluated.

### 3.2 Bleeding risk assessment during anticoagulation therapy for VTE

#### 3.2.1 HAS-BLED

Originally developed for AF patients, the HAS-BLED has also been studied in VTE patients. A retrospective study by Riva et al. (28) included 681 adult patients receiving warfarin for secondary VTE prevention. Among eight bleeding risk scores compared, HAS-BLED was the best predictor of clinically relevant bleeding (major bleeding and clinically relevant non-major bleeding) during the first three months of anticoagulation. In the first three months, the incidence of clinically relevant bleeding was 2.1% (95% CI: 0.5%–6.4%) in the low-risk group, 7.5% (95% CI: 5.1%–11.0%) in the intermediate-risk group, and 12.2% (95% CI: 7.6%–18.8%) in the high-risk group. The incidence of major bleeding in these groups was 0.7%, 2.0%, and 2.7%, respectively. However, when used solely for predicting major bleeding, the performance of all scores was suboptimal.

Another study indicated that a HAS-BLED score ≥3 was associated with increased major bleeding risk. Each controlled bleeding risk factor could reduce the risk of major bleeding by 20%–30%. However, during the first six months of VTE treatment, the major bleeding rate in the high-risk group varied significantly (2.4%–9.6%) (29). Although HAS-BLED has good validation for major bleeding, the variable “unstable INR” is less applicable to extended VTE anticoagulation. Replacing it with “active malignancy” may improve its relevance to VTE patients (30).

#### 3.2.2 Kuijer

The Kuijer was one of the earliest bleeding risk models for VTE patients on anticoagulation. It was developed to assess bleeding risk in VTE patients treated with vitamin K antagonists and/or heparin (31). This model included only three risk factors: age ≥60 years (1.6 points), female sex (1.3 points), and malignancy (2.2 points). A total score of ≥3 indicates high bleeding risk, 1–3 points indicate moderate risk, and 0 points indicate low risk. The Kuijer was advantageous due to its simplicity and ease of application, but it lacked comprehensive risk factor coverage.

A study by Keller et al. (32) analyzed the predictive value of the Kuijer in 1,204,895 hospitalized VTE patients in Germany between 2005 and 2017. The study classified 176,723 (14.7%) patients as low risk, 914,964 (75.9%) as moderate risk, and 113,208 (9.4%) as high risk. A higher Kuijer risk level was predictive of in-hospital mortality, major adverse cardiovascular events, intracranial hemorrhage, gastrointestinal bleeding, and the need for transfusion. Despite its large sample size, the study's retrospective nature presents limitations.

#### 3.2.3 ACCP

The American College of Chest Physicians (ACCP) was based on 18 bleeding risk factors and was designed to predict major bleeding risk. Its advantage lies in its broad risk factor coverage and reasonable accuracy in validation studies (33). However, it has limited predictive ability for bleeding during extended anticoagulation. A prospective observational cohort study assessed the ACCP in 2,263 patients undergoing long-term anticoagulation (1,522 on vitamin K antagonists and the rest on direct oral anticoagulants). Over 50% of patients were classified as high risk, with an annual bleeding rate of 1.7% vs. 0.95% (*P* = 0.05) in the low-risk group. The C-statistics for low, intermediate, and high risk groups were 0.55, 0.50, and 0.56, respectively. The study concluded that the ACCP had limited predictive value for bleeding events beyond 90 days of anticoagulation and was not suitable for guiding extended anticoagulation decisions (34).

#### 3.2.4 RIETE

The RIETE was developed based on a cohort of 19,274 patients with acute venous thromboembolism (VTE) receiving anticoagulation therapy within the first three months (35). The study included patients from 123 hospitals, with 13,057 (67%) in the modeling cohort and 6,572 in the validation cohort. The anticoagulation regimens consisted of initial low-molecular-weight heparin (LMWH) or heparin, followed by long-term LMWH or vitamin K antagonists (VKAs). The analysis identified six risk factors associated with major bleeding: recent major bleeding, renal impairment, anemia, malignancy, symptomatic pulmonary embolism, and age >75 years. The score stratified patients into low, intermediate, and high-risk groups for major bleeding. The incidence of major bleeding in the low, intermediate, and high-risk groups was 0.1%, 2.8%, and 6.2%, respectively (*P* < 0.05).

The limitations of the RIETE included its short follow-up period and the exclusion of patients receiving direct oral anticoagulants (DOACs). Subsequent external validation studies have demonstrated the score's moderate to poor predictive ability, particularly in Chinese populations, where its discriminatory performance was limited (36). However, a RIETE score >4 points has shown some predictive value for clinically relevant bleeding events during hospitalization, with a sensitivity of 60% and a specificity of 80% (37).

#### 3.2.5 OBRI

The outpatient bleeding risk index (OBRI) was used to assess bleeding risk in outpatients and consists of four factors: age ≥65 years, history of stroke, history of gastrointestinal ulcer bleeding, and the presence of any of the following comorbidities: recent myocardial infarction, hematocrit <30%, creatinine >1.5 mg/L, or history of diabetes. Each factor was assigned 1 point. Patients were classified into low (0 points), intermediate (1–2 points), and high-risk groups (≥3 points). The OBRI was initially derived from a retrospective study involving 543 patients with atrial fibrillation receiving warfarin for stroke prevention (38). In this study, 3.3% of patients experienced major bleeding, with a significantly higher incidence in the high-risk group compared to the low-risk group (*P* < 0.001). However, the OBRI was unable to predict minor bleeding events. Subsequent prospective validation in VTE patients demonstrated that the OBRI score effectively identifies VTE patients at low and intermediate bleeding risk, suggesting its broad applicability for VTE populations (39).

#### 3.2.6 EINSTEIN

This model was developed based on data from two large randomized controlled trials (RCTs): EINSTEIN-DVT and EINSTEIN-PE. The model used Cox proportional hazards regression with optimal subset variable selection to identify factors associated with major bleeding events (40). It considered three time windows: the first three weeks, after the third week, and the entire anticoagulation period. The model's discriminative ability was assessed using the C-statistic and internally validated with bootstrap techniques.

Among 4,130 patients treated with rivaroxaban, 40 (1.0%) experienced major bleeding, compared to 72 (1.7%) of the 4,116 patients treated with enoxaparin/VKAs. Notably, 44% of major bleeding events occurred within the first three weeks of treatment. The significant risk factors for major bleeding included older age, Black race, low hemoglobin levels, active malignancy, and concurrent antiplatelet or nonsteroidal anti-inflammatory drug (NSAID) therapy. The model demonstrated good discriminatory performance for major bleeding across the three time windows: first three weeks (C-statistic = 0.73), after three weeks (C-statistic = 0.68) and entire anticoagulation period (C-statistic = 0.74). While the EINSTEIN model identified bleeding risk factors in VTE patients receiving rivaroxaban or enoxaparin/VKAs, it lacked external validation and is not widely used in clinical practice.

#### 3.2.7 Hokusai

The Hokusai was derived from the Hokusai-VTE trial, which compared edoxaban with VKAs in 8,240 patients. It consisted of five risk factors: female sex, concurrent antiplatelet therapy, hemoglobin ≤100 g/L, history of hypertension, and systolic blood pressure >160 mmHg. The Hokusai score was simple, including only six variables with reasonable weighting, making it easy to use in clinical practice. In internal validation, it demonstrated better predictive performance compared to other scores (C-statistic = 0.71). However, in external validation, its predictive ability was weaker (C-statistic = 0.52), limiting its clinical applicability (41).

### 3.3 Bleeding risk assessment during extended anticoagulation therapy for VTE

#### 3.3.1 Seiler

The aforementioned bleeding risk score was primarily used to predict bleeding risk during the first three months of anticoagulation therapy, making them potentially unsuitable for extended anticoagulation. To address this, the Seiler was developed based on the SWITCO65+ study—a prospective cohort study of elderly VTE patients (aged ≥65 years). Researchers selected seven variables from 17 potential predictors: history of major bleeding (1 point), active malignancy (1 point), low physical activity (2 points), anemia (1 point), thrombocytopenia (1 point), use of antiplatelet agents or nonsteroidal anti-inflammatory drugs (NSAIDs) (1 point), and poor INR control (1 point). Patients were classified into three risk categories based on their total scores: low risk (0–1 points), intermediate risk (2–3 points), high risk (≥4 points). In the SWITCO65+ study, 743 VTE patients aged ≥65 years were categorized into low, intermediate, and high-risk groups and followed for 36 months during extended anticoagulation therapy. The incidence of major bleeding in the low, intermediate, and high-risk groups was 1.4, 5.0, and 12.2 events per 100 patient-years, respectively. The C-statistic of the Seiler for predicting bleeding risk at 3 and 36 months was 0.78 and 0.71, respectively, indicating good predictive performance. Compared with other scores (OBRI, Kuijer, and RIETE), the Seiler had higher C-statistics at 3, 6, 12, 24, and 36 months, demonstrating superior predictive ability (42).

In a subsequent analysis, Frei et al. (43) compared the predictive performance of 10 clinical bleeding risk scores (VTE-BLEED, Seiler, Kuijer, Kearon, RIETE, ACCP, OBRI, HEMORR_2_HAGES, HAS-BLED, and ATRIA) using the same cohort. The median anticoagulation duration was 10.1 months. The positive likelihood ratio (PLR) for predicting major bleeding ranged from 0.69 (OBRI) to 2.56 (Seiler), while the PLR for clinically relevant bleeding ranged from 1.07 (ACCP) to 2.36 (Seiler). Among the 10 scores, only five (Seiler, Kuijer, RIETE, HEMORR_2_HAGES, and ATRIA) identified a high-risk group with an annual bleeding rate of 6.5%. Notably, only two scores (Seiler and RIETE) had a PLR >2 for predicting major bleeding, highlighting their superior predictive accuracy. Overall, this study concluded that the Seiler score was the only acceptable model for predicting major bleeding in elderly patients undergoing extended anticoagulation therapy.

#### 3.3.2 VTE-BLEED

The VTE-BLEED was developed based on data from the RECOVER (dabigatran vs. warfarin, *n* = 2,539) and RECOVER II (dabigatran vs. warfarin, *n* = 2,589) trials. It was specifically designed to identify patients at high bleeding risk during stable oral anticoagulation therapy (i.e., more than 30 days after acute VTE). The score includes six risk factors: active malignancy (2 points), uncontrolled blood pressure in males (1 point), anemia (1.5 points), history of bleeding (1.5 points), renal impairment (creatinine clearance 30–60 ml/min) (1.5 points), age ≥60 years (1.5 points). A total score of ≥2 points indicated high bleeding risk.

The VTE-BLEED has been tested and validated in two phase III NOAC trials, as well as in a cohort study and a registry of patients receiving NOACs or VKAs. It demonstrated good discriminatory performance across different risk groups. The COMMAND VTE study compared the five-year cumulative incidence of major bleeding in high- and low-risk groups based on the VTE-BLEED. During stable anticoagulation with either VKAs or NOACs, the five-year cumulative incidence of major bleeding was significantly higher in the high-risk group compared to the low-risk group (13.2% vs. 5.4%, *P* < 0.001). The score also effectively identified patients at high risk of intracranial hemorrhage (ICH) or fatal bleeding, with a five-year cumulative incidence of 4.1% in the high-risk group vs. 1.4% in the low-risk group (44).

Given the variety of bleeding risk models applicable to VTE patients across different clinical scenarios (prophylaxis, acute treatment, and extended therapy), their distinctive features were consolidated for direct comparison in Table 3.

**Table 3:** Summary of bleeding risk assessment models in venous thromboembolism (VTE) patients.

Model	Original study design/validation cohort	Sample size (*n*)	Main predictive factors	Risk stratification	Major bleeding incidence (%)	C-statistic (AUC)	Key features
IMPROVE	International observational study of medical inpatients (derivation/validation split)	10,866 (derivation + validation)	Active gastroduodenal ulcer, prior bleeding, platelet count low, age, liver/renal failure, ICU stay, central venous catheter, malignancy, male sex	High risk: ≥7 points	0.76% (major) 1.4% (non-major but clinically relevant)	0.62–0.73	First bleeding risk model for VTE prevention; moderate to good discriminatory ability in medical inpatients
HAS-BLED	Originally AF warfarin cohorts; subsequently tested in VTE cohorts (retrospective/registry studies)	Variable by study (e.g., Riva et al. *n* = 681 for VTE secondary prevention)	Hypertension, abnormal renal/liver function, stroke, bleeding history, labile INR, elderly, drugs/alcohol	Low risk: 0–2 points Intermediate: 3 points High risk: ≥4 points	OAC: 1.75% Aspirin/Clopidogrel: 0.97% Neither: 1.42%	OAC: 0.72 Aspirin/Clopidogrel: 0.91 Neither: 0.85	Originally developed for AF; adapted for VTE with malignancy substitution
Kuijer	Early model derived from VTE patients treated with VKAs/heparin; small derivation cohort	Test group: 241, Validation group: 780	Age ≥60, female sex, malignancy	Low risk: 0 points Intermediate: 1–2 points High risk: ≥3 points	Test group: 4.0% Validation group: 2.0%	0.82	One of the earliest VTE bleeding risk models; simple three-factor model
ACCP	Score compiled from literature/expert consensus (18 risk factors); validated in cohorts	2,263 in one prospective long-term anticoagulation cohort	Composite of 18 bleeding risk factors (age, prior bleed, malignancy, renal failure, etc.)	Low risk: 0 points Intermediate: 1 points High risk: ≥2 points	Low risk:1.6% (0–3 months), 0.8% (≥3months); Intermediate: 3.2% (0–3 months), 1.6% (≥3months); High risk: 12.8% (0–3 months), 6.5% (≥3months);	0.50–0.56	Broad risk factor coverage but limited predictive value beyond 90 days
RIETE	Multicenter registry analysis (derivation and validation samples from 123 hospitals)	19,629 acute VTE (13,057 derivation; 6,572 validation)	Recent major bleeding, abnormal renal function, anemia, active cancer, symptomatic PE, age >75	Low risk:0 points Intermediate:1–4 points High risk: ≥4 points	Low risk: 0.1% Intermediate: 2.8% High risk: 6.2%	N/A	Derived from large international registry (19,274 patients); specifically designed for acute VTE phase; short-term follow-up focus; excludes DOAC patients
OBRI	Derived in warfarin-treated outpatient cohort; later prospectively validated in other cohorts	Warfarin: 565	Age ≥65, prior stroke, prior GI ulcer/bleed, and any of recent MI, Hct <30%, Cr >1.5 mg/dl, diabetes	Low risk: 0 points Intermediate: 1–4 points High risk: ≥4 points	12.0%	N/A	Effective for identifying low and intermediate bleeding risk in VTE populations
EINSTEIN	Developed from EINSTEIN DVT/PE randomized trials (rivaroxaban vs. enoxaparin/VKA) using Cox regression	Rivaroxaban: 4,130; Enoxaparin/ VKAs: 4,116	Age, race, low hemoglobin, active cancer, concomitant antiplatelet/NSAID use	Time-dependent risk assessment	Rivaroxaban:1.0% Enoxaparin/VKAs: 1.7%	0.68–0.74	Good discriminatory performance across different time windows; lacks external validation
Hokusai	Derived from Hokusai-VTE RCT (edoxaban vs. VKA; *n* = 8,240)	Edoxaban: 4,118 Warfarin: 4,122	Female sex, concomitant antiplatelet use, Hb ≤100 g/L, hypertension history, systolic BP >160 mmHg (and others)	Continuous risk scoring: 0–5 points	Edoxaban: 0 point (0.38%), 1point (1.01%), 2 points (2.1%), ≥3 points (5.4%); Warfarin: 0 point (1.1%), 1 point (1.4%), 2 points (2.1%), ≥3 points (3.7%)	Hokusai: 0.71 Kuijer: 0.62 Has-bled: 0.55, ORBIT: 0.61 EINSTEIN: 0.69	Simple variables but poor external validation limits clinical applicability
Seiler	Prospective cohort of elderly VTE patients (SWITCO65 + study)	VKA: 743 (age ≥65)	Prior major bleed, active cancer, low physical activity, anemia, thrombocytopenia, antiplatelet/NSAID use, poor INR control	Low risk: 0–1 points Intermediate: 2–3 points High risk: ≥4 points	Low risk: 15.0% Intermediate: 37.0% High risk: 48.0%	3-month: 0.78 36-month: 0.71	Specifically designed for extended anticoagulation in elderly; superior to other scores
VTE-BLEED	Derived from pooled RECOVER and RE-COVER II trial data	Dabigatran: 2,553 Warfarin: 2,554	Active cancer, male with uncontrolled hypertension, anemia, prior bleeding, CrCl 30–60 ml/min, age ≥60	Low risk: 0–1.5 points High risk: ≥2 points	Low risk: 5.4% (major bleeding), 1.4% (fatal bleeding) High risk: 13.2% (major bleeding), 4.1% (fatal bleeding)	0.72	Identifies high-risk patients for intracranial/fatal bleeding during long-term therapy

N/A, indicates that specific values were not provided in the referenced studies.

### 3.4 Comparison of VTE bleeding risk models

Current VTE guidelines recommended that clinicians selected their preferred bleeding risk assessment tool, as no specific score was universally endorsed. Most guidelines do not specify a particular bleeding risk model. However, the 2020 NICE VTE guidelines recommended using the HAS-BLED to assess bleeding risk. If the score is ≥4 and reversible factors cannot be corrected, anticoagulation therapy should be discontinued (45). The ISTH guidelines did not recommend using VTE bleeding risk models as standalone tools to determine the optimal treatment duration. Instead, they suggested using these models to predict bleeding risk during extended anticoagulation and to guide the frequency of follow-up assessments. Patients with higher bleeding risk may benefit from more frequent reassessments (3). Among the various bleeding risk models, VTE-BLEED, RIETE, and ACCP have undergone external validation in diverse populations, including patients receiving both VKAs and NOACs. Therefore, these models are recommended for long-term follow-up of VTE patients due to their broader validation base.

## 4 Bleeding risk assessment models for special populations

### 4.1 Bleeding risk assessment in cancer patients

Patients with cancer-associated venous thromboembolism (CAVTE) faced significantly increased risks of both recurrence and bleeding compared to non-cancer patients. The 2020 CSCO guidelines recommended considering prophylactic anticoagulation for all patients with active malignancies, particularly those undergoing chemotherapy. Following anticoagulation, the risk of major bleeding in CAVTE patients was 2–6 times higher than in non-cancer patients, while the risk of VTE recurrence was approximately 3 times higher (46–48). Therefore, achieving a balance between thrombosis prevention and bleeding risk was crucial, requiring early identification of high-risk patients and the establishment of comprehensive risk assessment systems.

For CAVTE patients, the IMPROVE can be used to evaluate bleeding risk, while for atrial fibrillation (AF) patients with cancer, no specific guideline-recommended bleeding risk model is currently available. A retrospective cohort study involving 399,344 AF patients with cancer evaluated the predictive performance of the HAS-BLED, ATRIA, and ORBIT (49). Over a mean follow-up of two years, the annual incidence rates of major bleeding, gastrointestinal (GI) bleeding, and intracranial hemorrhage (ICH) were 8.41%, 3.61%, and 1.33%, respectively. The most common sites of major bleeding included the liver (12.68%/year), leukemia-related bleeding (12.39%/year), pancreas (11.71%/year), bladder (11.67%/year), and multiple myeloma (11.64%/year). GI bleeding was most frequently observed in the liver (7.54%/year), pancreas (7.42%/year), and stomach (5.51%/year), while ICH was most common in patients with leukemia (1.89%/year), multiple myeloma (1.52%/year), lymphoma and liver cancer (1.45%/year), and pancreatic cancer (1.41%/year). The study found that all three scoring models were significantly associated with major bleeding, GI bleeding, and ICH. Among them, the HAS-BLED performed best in predicting ICH, while the ORBIT showed the highest accuracy in predicting both major and GI bleeding.

Nevertheless, accumulating evidence suggests that traditional bleeding risk models (e.g., HAS-BLED, ORBIT) have limited predictive value in cancer patients, with discrimination (AUC) frequently falling below 0.6—considerably lower than in non-cancer populations. This reduced performance largely results from the omission of cancer-specific risk determinants such as tumor type, stage, site, degree of bone marrow suppression, and the hematologic and vascular effects of antineoplastic therapies (e.g., chemotherapy, targeted agents, surgery). Consequently, the predictive results of conventional models should be interpreted with caution in oncology patients, as they may substantially underestimate bleeding risk. A validation study by Ajabnoor et al. (50) validated the HAS-BLED in 141,796 AF patients, of whom 10.3% had cancer, including breast, prostate, colorectal, lung, and hematologic cancers. The HAS-BLED had poor discriminatory ability in the cancer cohort (AUC < 0.6) but showed moderate discrimination in the non-cancer cohort (AUC = 0.61), indicating that its predictive performance may be limited in cancer patients.

Taken together, these findings highlight an urgent need for cancer-specific bleeding risk models that integrate oncologic variables, hematologic indices, and treatment-related factors. Future studies leveraging large cancer registries and real-world datasets should aim to construct and validate such specialized tools to enable individualized anticoagulation management in this complex, high-risk population.

### 4.2 Gastrointestinal bleeding risk assessment

Currently, several models, such as the Forrest classification, Rockall bleeding and mortality risk score, Glasgow-Blatchford score (GBS), and The Baylor bleeding risk score, were widely used to assess the risk of recurrent bleeding, intervention needs, and mortality in patients with existing upper GI bleeding. However, these models may not be suitable for assessing the risk of anticoagulation-related GI bleeding. To address this gap, Lv et al. (12) developed the Alfalfa-DOAC-GIB, specifically designed to predict major GI bleeding risk in patients receiving NOAC therapy. The study included 11,903 patients from 22 medical centers, using logistic regression to establish the model and comparing it with the HAS-BLED.

The Alfalfa-DOAC-GIB incorporated 12 risk factors, including age ≥65 years, alcohol consumption, history of peptic ulcer, prior major bleeding, liver and kidney dysfunction, malignancy, low platelet count, anemia, concomitant use of antiplatelet agents, NSAIDs, and gastroprotective agents. A total score ≥3.5 indicated high bleeding risk. Internal validation using the bootstrap method demonstrated that the Alfalfa-DOAC-GIB outperformed the HAS-BLED in predicting GI bleeding risk (AUC = 0.778 vs. 0.69), making it a more effective tool for identifying high-risk patients.

Clinically, these findings emphasize that HAS-BLED is not the optimal tool for evaluating gastrointestinal bleeding risk in patients treated with NOACs. In contrast, the Alfalfa-DOAC-GIB provides better discrimination and should be considered when available to identify patients who may benefit from preventive interventions, such as proton pump inhibitor (PPI) prophylaxis or careful drug adjustment, to mitigate GI bleeding risk (12). Consequently, the incorporation of GI-specific models represents a crucial step toward precision anticoagulation management and improved patient safety in clinical practice.

### 4.3 Intracranial hemorrhage risk assessment

For patients with acute ischemic stroke (AIS) undergoing thrombolysis, no specific bleeding risk assessment model was currently recommended in guidelines. However, several models were commonly used to predict intracranial hemorrhage (ICH) risk.

The HAT includes NIHSS, diabetes history or baseline blood glucose, and early CT signs of hypodensity, with a total score range of 0–5 points. The risk of symptomatic ICH increases with higher scores, ranging from 2% at 0 points to 44% at >3 points. The HAT model was simple, easy to use, and facilitates rapid bedside assessment (C-statistic = 0.72), but its predictive accuracy for patients treated outside the standard 3-hour thrombolysis window remained uncertain.

The SITS considers age, NIHSS, blood glucose, prior antithrombotic use, systolic blood pressure (SBP), body weight, time from symptom onset to thrombolysis, and history of hypertension, with a total score range of 0–12 points. A score ≤2 corresponds to an estimated 0.4% risk of symptomatic ICH, while a score ≥10 indicates a 9.2% risk. The model has broad applicability, demonstrating good predictive accuracy (C-statistic = 0.70).

The SEDAN, which included age, NIHSS score, baseline blood glucose, early CT hypodensity, and hyperdense MCA sign, provided good predictive value for both anterior and posterior circulation AIS. However, it does not account for blood pressure fluctuations, a factor that may significantly impact bleeding risk.

The GRASPS consists of age, baseline NIHSS score, SBP, blood glucose, race, and gender, with a total score range of 0–101 points. It was unique in incorporating racial differences as a risk factor but did not include imaging findings.

Overall, each of these models has strengths and limitations. While they can assist clinicians in assessing the risks and benefits of thrombolysis and guiding personalized treatment strategies, they should not be used as standalone tools to exclude patients from thrombolysis or retrospectively evaluate whether a patient should have undergone the procedure.

## 5 Conclusion

Extensive research has been conducted worldwide on bleeding risk assessment models, with many demonstrating moderate to good predictive performance. However, the heterogeneity in study design, patient populations, anticoagulation regimens, follow-up durations, definitions of major bleeding, and included risk factors has resulted in varying prognostic endpoints and applicability across different models. Substantial heterogeneity in endpoint definitions across studies, particularly between the International Society on Thrombosis and Haemostasis (ISTH) and Thrombolysis in Myocardial Infarction (TIMI) bleeding criteria, poses an additional challenge for cross-study comparisons. These differing definitions of “major bleeding” and “clinically relevant non-major bleeding” can lead to inconsistent classification of outcomes, thus affecting both the derivation and validation of risk prediction models. Harmonization of bleeding criteria across clinical studies is therefore essential for improving model comparability, external validation, and integration into guideline-based clinical practice.

It is also important to recognize that most classical bleeding risk assessment models were developed in the warfarin-associated models, when patient monitoring, pharmacokinetics, and therapeutic intensity differed markedly from current non–vitamin K antagonist oral anticoagulant (NOAC) regimens. Consequently, their performance in modern clinical practice may be suboptimal. The direct “transplantation” of these models into NOAC-treated populations raises concerns regarding their predictive accuracy and clinical relevance. Recalibration and external validation in contemporary cohorts are therefore necessary to enhance model precision and applicability.

Another critical limitation shared by most current models is their reliance on static baseline variables, which fail to capture dynamic changes in patient condition throughout treatment. In real-world settings, factors such as transient anemia, fluctuating renal function, and concomitant antiplatelet therapy can significantly alter bleeding risk over time. Future risk assessment models should incorporate time-varying predictors and adopt adaptive, machine-learning–based frameworks capable of real-time recalibration, thereby improving individualized bleeding risk prediction and supporting precision anticoagulation management.

Despite methodological differences, most bleeding risk scores identified five common high-risk factors: advanced age, malignancy, history of bleeding, renal insufficiency, and anemia. The 10th edition of the ACCP guidelines does not endorse a single preferred bleeding risk score but instead highlights a list of key risk factors, including age over 65 years, previous bleeding, malignancy, stroke, recent surgery, concomitant use of antiplatelet agents or NSAIDs, renal and hepatic dysfunction, thrombocytopenia, anemia, diabetes, poor anticoagulation control, functional decline, frequent falls, and heavy alcohol consumption (more than eight drinks per week). Patients with two or more of these risk factors were classified as high risk, with an estimated bleeding incidence of exceeding 6.5%.

To minimize bleeding risk, early identification of high-risk patients and proactive intervention are essential. Addressing modifiable risk factors can significantly reduce bleeding incidence. Key measures include correcting anemia, stabilizing blood pressure, and optimizing liver and kidney function. Patients receiving NSAIDs or antiplatelet therapy should be considered for proton pump inhibitor (PPI) prophylaxis to reduce gastrointestinal bleeding risk. Patient education is also critical; for example, warfarin users should receive enhanced monitoring of INR levels, with appropriate adjustments based on thrombosis and bleeding risk. For patients at risk of falls, neurological screening should be conducted, and preventive strategies such as anti-slip footwear or mobility aids should be implemented. Additionally, alcohol cessation counseling should be provided for individuals with heavy alcohol consumption, and smoking cessation support should be encouraged.

Despite advancements in bleeding risk prediction, further research is needed to conduct large-scale, multicenter, real-world prospective studies to refine risk stratification in AF and VTE patients. Additionally, validation of bleeding risk models across diverse ethnicities and geographic regions is necessary to improve generalizability and clinical applicability. Future efforts should focus on enhancing predictive algorithms, integrating biomarker-based and dynamic risk assessment approaches, and leveraging artificial intelligence to develop more personalized, adaptive, and clinically actionable bleeding risk models. Such advancements will ultimately strengthen precision anticoagulation management, reduce bleeding complications, and improve long-term outcomes and quality of life for patients requiring anticoagulant therapy.
